# Genetic variability in E6, E7 and L1 genes of Human Papillomavirus 62 and its prevalence in Mexico

**DOI:** 10.1186/s13027-017-0125-x

**Published:** 2017-03-04

**Authors:** Cristina Artaza-Irigaray, María Guadalupe Flores-Miramontes, Dominik Olszewski, María Teresa Magaña-Torres, María Guadalupe López-Cardona, Yelda Aurora Leal-Herrera, Patricia Piña-Sánchez, Luis Felipe Jave-Suárez, Adriana Aguilar-Lemarroy

**Affiliations:** 1 División de Inmunología, Centro de Investigación Biomédica de Occidente (CIBO), Instituto Mexicano del Seguro Social (IMSS), Guadalajara, Jalisco Mexico; 2 Programa de Doctorado en Ciencias Biomédicas, Centro Universitario de Ciencias de la Salud (CUCS), Universidad de Guadalajara, Jalisco, Mexico; 3 Institute of Pharmacy and Molecular Biotechnology, University of Heidelberg, Heidelberg, Germany; 4 Unidad de Medicina Genómica y Genética, Hospital Regional Dr. Valentín Gómez Farías - ISSSTE, Guadalajara, Jalisco Mexico; 5 Unidad de Investigación Médica Yucatán (UIMY) - IMSS, Mérida, Yucatán Mexico; 6 Laboratorio de Oncología Molecular, Unidad de Investigación Médica en Enfermedades Oncológicas (UIMEO) - IMSS, Ciudad de Mexico, Mexico

**Keywords:** Cervical cancer, HPV62, E6, E7, L1

## Abstract

**Background:**

Human papillomavirus (HPV) is the main etiological agent of cervical cancer, the third most common cancer among women globally and the second most frequent in Mexico. Persistent infection with high-risk HPV genotypes is associated with premalignant lesions and cervical cancer development. HPVs considered as low risk or not yet classified, are often found in coinfection with different HPV genotypes. Indeed, HPV62 is one of the most prevalent HPV detected in some countries, but there is limited information about its prevalence in other regions and there are no HPV62 variants currently described. The aim of this study was to determine the prevalence of HPV62 in cervical samples from Mexican women and to identify mutations in the L1, E6 and E7 genes, which have never been reported in our population.

**Methods:**

HPV screening was performed by Cobas HPV Test in women who attended prevention health programs and dysplasia clinics. All HPV positive samples (*n* = 491) and 87 additional cervical cancer samples were then genotyped with Linear Array HPV Genotyping test. Some samples were selected to corroborate genotyping by Next-Generation sequencing. On the other hand, nucleotide changes in L1, E6 and E7 genes were determined using PCR, Sanger sequencing and analysis with the CLC-MainWorkbench 7.6.1 software. L1 protein structure was predicted with the I-TASSER server.

**Results:**

Using Linear Array, HPV62 prevalence was 7.6% in general population, 8% in Cervical Intraepithelial Neoplasia grade 1 (CIN1) samples and 4.6% in cervical samples. The presence of HPV62 was confirmed with Next-Generation sequencing. Regarding L1 gene, novel sequence variations were detected, but they did not alter the tertiary structure of the protein. Moreover, several nucleotide substitutions were found in E6 and E7 genes compared to reference HPV62 genomic sequence. Specifically, three non-synonymous sequence variations were detected, two in E6 and one in E7.

**Conclusions:**

HPV62 is a frequent HPV genotype found mainly in general population and in women with CIN1, and in 90.5% of the cases it was found in coinfection with other HPVs. Novel nucleotide changes in its *L1, E6* and *E7* genes were detected, some of them lead to changes in the protein sequence.

## Background

Cervical Cancer (CC) is the fourth leading cause of cancer deaths in women worldwide with an estimate of more than 528,000 new diagnosed cases and 266,000 deaths in 2012. More than 85% of deaths occur in developing countries and in particular, in Mexico, CC is the second most frequent cancer leading to 4,769 deaths in 2012 [1]. This pathology is directly associated with HPV (Human Papillomavirus) infection and to date, around 200 HPVs have been described [2, 3]. Mucosal HPVs are grouped into low risk (LR-HPVs) and high risk HPVs (HR-HPVs), the latter being considered the etiologic agents of CC [3]. The *Alphapapillomavirus* genus harbors more than 60 types of HPVs, including the oncogenic HPVs associated to anogenital cancers according to the International Agency for Research on Cancer (IARC): types 16, 18, 31, 33, 35, 39, 45, 51, 52, 56, 58, 59, 66 and 68 [4, 5]. Specifically, HPV16 and HPV18 are found in around 70% of the CC cases worldwide [6, 7].

HPVs have a circular double-stranded 8 kb DNA genome that typically contains eight genes [8]. The L1 gene, which encodes the principal virus capsid protein, is used for the classification and construction of phylogenetic trees as it is well conserved among different HPVs. In 2004, the HPV classification criteria were defined based on differences in the complete L1 Open Reading Frame (ORF): different genera share less than 60% nucleotide sequence identity, HPV species within a genus share between 60 and 70% identity, HPV types share between 71 and 89% nucleotide identity, HPV subtypes differ in 2-10% and HPV variants differ in 1-2% within the L1 ORF [9]. In 2013, the term variant was proposed to also include HPV subtypes. The use of full genome sequence information, instead of the L1 ORF, was recommended to classify a new variant genome. The alignment of complete viral genomes began to define variant lineages and sublineages using differences of 1-10% and 0.5-1%, respectively [10].

HR-HPV genomes encode three oncoproteins ─E5, E6 and E7─ that contribute to enhanced cell proliferation, initiation and progression of CC [11]. An interesting review contrasts the activities of the human alpha-PV oncoproteins with their non-oncogenic counterparts based on cell culture studies [12]. The comparison of activities of LR- and HR-HPVs would lead to the identification of common activities probably needed for the viral life cycle, while additional functions of HR-HPVs could be crucial for the transformation/immortalization process.

HPV62 was characterized in 2004 by Fu et al. (accession number AY395706) from a cervical sample obtained from a 45-year-old woman with normal cytology [13] and it is considered as a LR-HPV. The E6 (447 bp), E7 (291 bp) and L1 (1512 bp) genes from this HPV62 reference genome (8092 bp) encode for 148, 96 and 503 amino acid proteins, respectively. In a first report including cervical samples from mexican population, HPV62 was mainly detected in coinfection with other HPV genotypes and it was found in 5.1% of HPV positive patients with Cervical Intraepithelial Neoplasia Grade 1 (CIN1) and in 0.8% of HPV positive patients with CC using Linear Array HPV Genotyping test [14]. The aim of this study, was to determine the prevalence of this genotype in a greater number of samples among Mexican women and to detect possible mutations in L1, E6 and E7 genes of the HPV62 circulating in the Mexican population. Until now, only two complete genome sequences have been reported worldwide from 2 patients: the first one in 2004 (AY395706) [13] and the second one very recently uploaded (KU298924.1) [15].

## Methods

### Sample collection

All samples were collected by gynecologists with a cytobrush inserted into the endocervical canal and placed into the transport medium PreservCyt (Hologic, Bedford, MA). Three large groups of samples were included: 1) cervical samples from women (general population), who attended cervical cancer prevention programs; 2) cervical samples with CIN1 from women who attended a Dysplasia Clinic, and 3) cervical samples with CC from women who attended the Oncology Hospital. The first group of samples include women from six different States of the Mexican Republic (Aguascalientes, Colima, Guanajuato, Jalisco, Michoacán, Nayarit and Yucatán), and they were obtained from the Regional Hospital Dr. Valentín Gómez Farías – ISSSTE (Guadalajara, Jalisco). The samples from the second group were recruited at the Dysplasia Clinic of the Regional General Hospital No. 12 Lic. Benito Juárez – IMSS (Mérida, Yucatán), and at the Dysplasia Clinic of the Western National Medical Center – IMSS (Guadalajara, Jalisco). Finally, the last group’s samples were obtained exclusively from the Oncology Hospital of the Western National Medical Center – IMSS (Guadalajara, Jalisco). In all cases, the samples were taken as part of the routine diagnosis confirmation and an aliquot of the PreservCyt solution was given for our study, after informed consent was signed. Samples with excess of blood and mucus in the PreservCyt solution, low DNA quantity or quality samples, were eliminated. A total of 2835 samples were screened for HPV; 2399 from general population, 349 from Dysplasia Clinics and 87 from Oncology Hospital. Concerning only the first group of samples, the diagnosis was kept anonymous for this study, as authorized in the ethically approved protocol. However, the diagnosis of the second (CIN1) and third (CC) group of samples was obtained by colposcopic observation and confirmed by histopathological analysis.

Samples were collected from July 2014 to July 2016. As detailed in the section of ethical considerations, collection of the samples from the different groups was authorized by the ISSSTE and the National Committee on Health Research and Ethics of the IMSS, for various research protocols.

### HPV screening and genotyping

The samples from the first and the second group were first screened for HPV positivity with the Cobas HPV Test (Roche Molecular Systems, Inc). Afterwards, all HPV positive samples were genotyped with the Linear Array HPV Genotyping test (LA), Roche Molecular Diagnostics. Additionally, 48 CIN1 samples taken randomly, were genotyped with the 454 Next-Generation Sequencing (NGS) platform to confirm HPV genotyping. Regarding the third group, all CC samples were genotyped with LA and 48 of them were also selected randomly to confirm genotyping with NGS. The set of PGMY11/09 primers (452 bp amplicon from nucleotide 949 to 1400 of L1 ORF) was used for NGS, as previously described [16]. In addition, NGS was also performed using the degenerated FAP primers set [17, 18]. A first conventional Polymerase Chain Reaction (PCR) was done with the primers FAP59 (forward 5′- TAACWGTIGGICAYCCWTATT - 3′) and FAP64 (reverse 5′-CCWATATCWVHCATITCICCATC - 3′) to amplify a 478 bp fragment from the L1 region. PCR conditions were: 94 °C for 3 min, 35 cycles at 94 °C for 45 s, 50 °C for 30 s and 72 °C for 1 min, finally 72 °C for 10 min. A second PCR was done with the 478 bp amplicon using the following primers: Forward 5′- [Universal Multiplicom tail A: AAGACTCGGCAGCATCTCCA] – [FAP6085: CCWGATCCHAATMRRTTTGC]-3′. and Reverse 5′ [Universal Multiplicom tail B: GCGATCGTCACTGTTCTCCA] – [FAP64 primer]. The resulting amplicon is of 377 bp (from nucleotide 232 to 607 of L1 ORF). Universal Multiplicom tails A and B were the same used in Cat. no. MR-0020.024 (Multiplicom NV CFTR; Molecular Diagnostics, Niel, Belgium). This second PCR was run under the same conditions as the first PCR, except for the annealing temperature that was changed to 47 °C. Amplicons were visualized by gel electrophoresis in a 1.5% agarose gel. HPV positive samples were selected to undergo further screening by NGS using Multiplex identifiers (MID) barcodes for each sample as described in Flores-Miramontes et al. [16]. Quality control of the obtained sequences was carried out with the online platform Galaxy (version 16.04) and they were analyzed with Roche′s GS Reference Mapper Software (version 2.9), using as references all human papillomavirus sequences from the Papillomavirus Episteme (PaVE) database [3, 19].

### Sanger sequencing for L1, E6 and E7 genes

E6, E7 and L1 genes were amplified from cervical samples positive to HPV62 by Linear Array using PCR with the following primer pairs specific for each gene of interest (all of them were designed with Oligo v6 software). E6: forward 5′- GGTCAGCACAGTAGCAATGACT-3′ and reverse 5′- CGGGACGCTCTTGTAGGAC- 3′; E7: forward 5′-CAGGAGTGTGGACAGGACGGTA- 3′ and reverse 5′- GCATCGGCCATGTCACTTATG -3′; L1: forward 5′- ACGCCTTCCTTCCCTGCAACTA - 3′ and reverse 5′-CACTGACAAACGCGCACAACAC-3′. The reactions were performed in a final volume of 25 uL containing at least 200 ng of genomic DNA, 200 uM of each dNTP, 1X reaction buffer with 1.8 mM of MgCl_2_, 12.5 pmol of each primer, and 1.25 units of Fast Start High Fidelity enzyme (Roche Applied Science, Cat. No. 04 738 284 001). PCR conditions were: initial denaturation at 95 °C for 2 min, 35 cycles of 95 °C for 30 s, annealing at 58 °C (*HPV62-E6* and *-E7*) or 62 °C (*HPV62-L1*) for 30 s, elongation at 72 °C for 45 s, and a final extension at 72 °C for 7 min. Afterwards, 5 uL of the PCR products were visualized on 1% agarose gel to corroborate the presence and size of the amplicon, and the other 20 uL were utilized for isopropanol purification. Purified amplicons were sequenced with BigDye Terminator v3.1 Cycle Sequencing Kit (Applied Biosystems, Cat. No. 4337455) using the above mentioned forward primers for E6, E7, and L1 and additional primers designed for L1, which is too long to be sequenced with a single primer (5′-ACACGGAACGCATGGTATGGGC, 5′-GCAGAACCTTATGGCGATTGTA-3′ and 5′-TTGTGCAAAATACAGTTAACCC-3′). Reactions were performed in a final volume of 20 uL with around 50 ng of DNA, 10 pmol of forward primer, 2 uL of 5X Sequencing Buffer, and 4 uL of Ready Reaction Premix. Cycling conditions were set as follows: initial denaturation at 96 °C for 1 min, and 25 cycles at 96 °C for 10 s, annealing at 50 °C for 5 s, elongation at 60 °C for 4 min. Finally, products were purified with Centri-Sep Spin Columns (ABI, Cat. No. 401762), and sequenced with the ABI PRISM 310 Genetic Analyzer (Applied Biosystems).

### Sequence alignments and L1 protein structure prediction analysis

To detect genetic mutations or variants in E6, E7 and L1, the obtained gene sequences were aligned to the HPV62 reference sequence (reported as AY395706 in the NCBI database) using the *CLC-MainWorkbench 7.6.1* program (Qiagen). To look for amino acid changes, the DNA sequences were translated into protein with the same software, and aligned to the reference protein. The phylogenetic tree showing alpha-3 group HPVs and all HPV62 sequences obtained in this work, was performed with MEGA v.7.014 software using Maximum Likelihood statistical method.

Finally, the L1 protein structures were predicted with the I-TASSER server (Iterative Threading ASSEmbly Refinement), which identifies structural templates from the Protein Data Bank by multiple threading approach and constructs full-length atomic models by iterative template fragment assembly simulations [20–23]. Protein structure alignment was performed with *CLC MainWorkbench 7.6.1* program.

## Results

### HPV62 prevalence genotyped by Linear Array HPV Genotyping Test

Regarding the first group of cervical samples described in “Methods section” (general population of women who attended cervical cancer prevention health programs), from the 2399 samples screened for HPV positivity by Cobas HPV Test, 291 were HPV positive (12.1%) and 22/291 (7.6%) were HPV62 positive. Concerning the second group (CIN1 samples), from the 349 screened with Cobas, 200 (57.3%) were HPV positive and 16/200 (8%) were HPV62 positive. Finally, the third group of samples (CC samples) showed 100% of HPV positivity with LA, and from those, only 4 samples (4.6%) were HPV62 positive. It is important to highlight that in only 4 samples from the total of 42 HPV62 positive samples, this genotype was found as single infection (exclusively in CIN1 samples), and in the other 38 (90.5%) in coinfection with 1 to 5 additional HPV genotypes (detailed in Table 1).

**Table 1:** HPV genotypes detected in coinfection with HPV62 by Linear Array in each of the 38 HPV62 positive samples

GROUP 1 SAMPLES (general population)			
Sample Code	HPV genotypes detected by Linear Array	Sample Code	HPV genotypes detected by Linear Array
GP-1	**16**, 42, **58**, 81, 83	GP-12	**39**, **59**, 61
GP-2	**31**, **52**, 61, 72, 84	GP-13	42, **45**, 84
GP-3	**39**, **51**, **56**, **58**, 84	GP-14	**16**, **18**
GP-4	11, **52**, **59**, 83	GP-15	**16**, **51**
GP-5	**31**, **39**, *66*, *67*	GP-16	**16**, **52**
GP-6	**39**, 61, 84, 89	GP-17	**31**, **59**
GP-7	**52**, **56**, *70*, 72	GP-18	**35**, *67*
GP-8	11, **16**, **39**	GP-19	**45**, 72
GP-9	**16**, **51**, **59**	GP-20	**51**, **59**
GP-10	*26*, **45**, *66*	GP-21	**58**, *66*
GP-11	**31**, 72, 83	GP-22	**51**
GROUP 2 SAMPLES (CIN1)			
Sample Code	HPV genotypes detected by Linear Array	Sample Code	HPV genotypes detected by Linear Array
CIN1-1	**33**, **45**, **59**, 71, 83	CIN1-7	**39**, 81
CIN1-2	**16**, **51**, **59,** 81	CIN1-8	**51**, *53*
CIN1-3	11, *70*, 83	CIN1-9	*70*, 83
CIN1-4	**16**, **39**, *66*	CIN1-10	**16**
CIN1-5	61, 72, 84	CIN1-11	**59**
CIN1-6	**16**, *53*	CIN1-12	89
GROUP 3 SAMPLES (CC)			
Sample Code	HPV genotypes detected by Linear Array	Sample Code	HPV genotypes detected by Linear Array
CC-1	**16**, 54, *70*	CC-3	**39**, 71
CC-2	**16**, **18**	CC-4	**16**

In bold: HPV genotypes classified as carcinogenic to humans by the IARC (group 1); in italics: possibly carcinogenic to humans (group 2B)

**Fig. 1:**
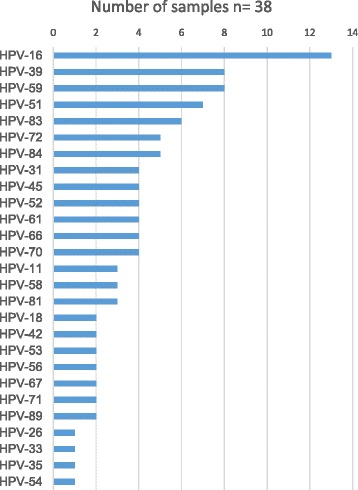
Frequency of HPV genotypes found in coinfection with HPV62. The graphic depicts the number of samples in which each HPV genotype was found together with HPV62

Interestingly, as depicted in Fig. 1, HPV62 was more frequently found in coinfection with 16, 39, 59, 51 and 83 HPV genotypes.

### HPV genotyping by NGS

To confirm HPV genotyping, 48 CIN1 samples were sequenced with NGS, 8 of them HPV62 positive with Linear Array. In those 8 samples, HPV62 presence was corroborated using NGS; however, 3 additional samples were positive to HPV62 only with the last methodology.

Concerning the presence of HPV62 exclusively in cervical cancer, 48 samples diagnosed with squamous cervical carcinoma were selected for HPV genotyping by NGS (choosing preferentially those that showed more than two HPV genotypes detected by Linear Array). To detect a broader HPV genotype spectrum in those samples, NGS was performed utilizing PGMY11/09 and FAP primer sets. As depicted in Table 2, four of the samples mapped with high identity to HPV62, and they were found in coinfection with additional HPV genotypes, including beta-1 papillomavirus (12, 21 and 118), which were only detected with FAP primers.

**Table 2:** HPV genotypes detected in samples from cervical cancer that were positive to HPV62 by NGS

Sample code	HPV types found by Linear Array	HPV types found by NGS	Reads using FAP primers	Reads using PGMY primers	Total Reads
CC-1	16, 54, 62, 70	16	-	174	174
		33	-	1	1
		54	776	3	779
		62	105	-	105
		70	163	348	511
		118	2	-	2
CC-2	16, 18, 62	16	9	481	490
		62	895	3	898
CC-3	39, 62, 71	16	-	50	50
		39	-	2	2
		62	1086	218	1304
		71	-	98	98
		81	2	-	2
CC-5	33	12	21	-	21
		21	1069	-	1069
		33	1	507	508
		62	3	-	3

Reads obtained from each HPV type by using FAP or PGMY11/09 primers are detailed for each sample. Linear Array results are also included

### Nucleotide variations in HPV62-L1

To study whether the HPV62 circulating in the Mexican population exhibits nucleotide changes in L1, all HPV62-L1 sequences obtained by NGS with PGMY or FAP primers (independently of the diagnosis of the samples) were aligned to the HPV62 complete genome (AY395706). The contigs were obtained from 11 samples amplified with PGMY primers and 9 samples amplified with FAP primers. Alignment of the eleven HPV62-L1 contigs revealed the presence of 9 mutations distributed among all the samples in the region amplified with PGMY primers. As depicted in Table 3, three out of the nine mutations were non-synonymous. Moreover, nine HPV62-L1 contigs from the region amplified with FAP primers revealed 10 mutations, five of them non-synonymous (Table 3).

**Table 3:** Genetic mutations found in the 5′ and 3′-ends of HPV62-L1

Nucleotide changes (PGMY primers)	Amino acid change	Number of samples with the mutation *n* = 11	Nucleotide changes (FAP primers)	Amino acid change	Number of samples with the mutation *n* = 9
c.969 T > C		2	c.250A > G	p.T84A	9
c.987A > C *	p.E329D	3	c.263C > G	p.A88G	7
c.1017G > A		1	c.265A > T	p.T89S	1
c.1071 T > C *		3	c.413C > T	p.A138V	1
c.1104G > A		1	c.436A > G	p.I146V	1
c.1236 T > C		3	c.438C > T		1
c.1256A > G	p.H419R	4	c.468A > G		1
c.1279A > G *	p.T427A	1	c.495A > T		5
c.1287A > G		3	c.495A > G		1
			c.516C > A		1

Nucleotide changes are shown in samples amplified with PGMY or FAP primers. The amino acid changes are described for those with non-synonymous mutations. The number of samples that carry each nucleotide change are included. (*) Already reported mutations

**Fig. 2:**
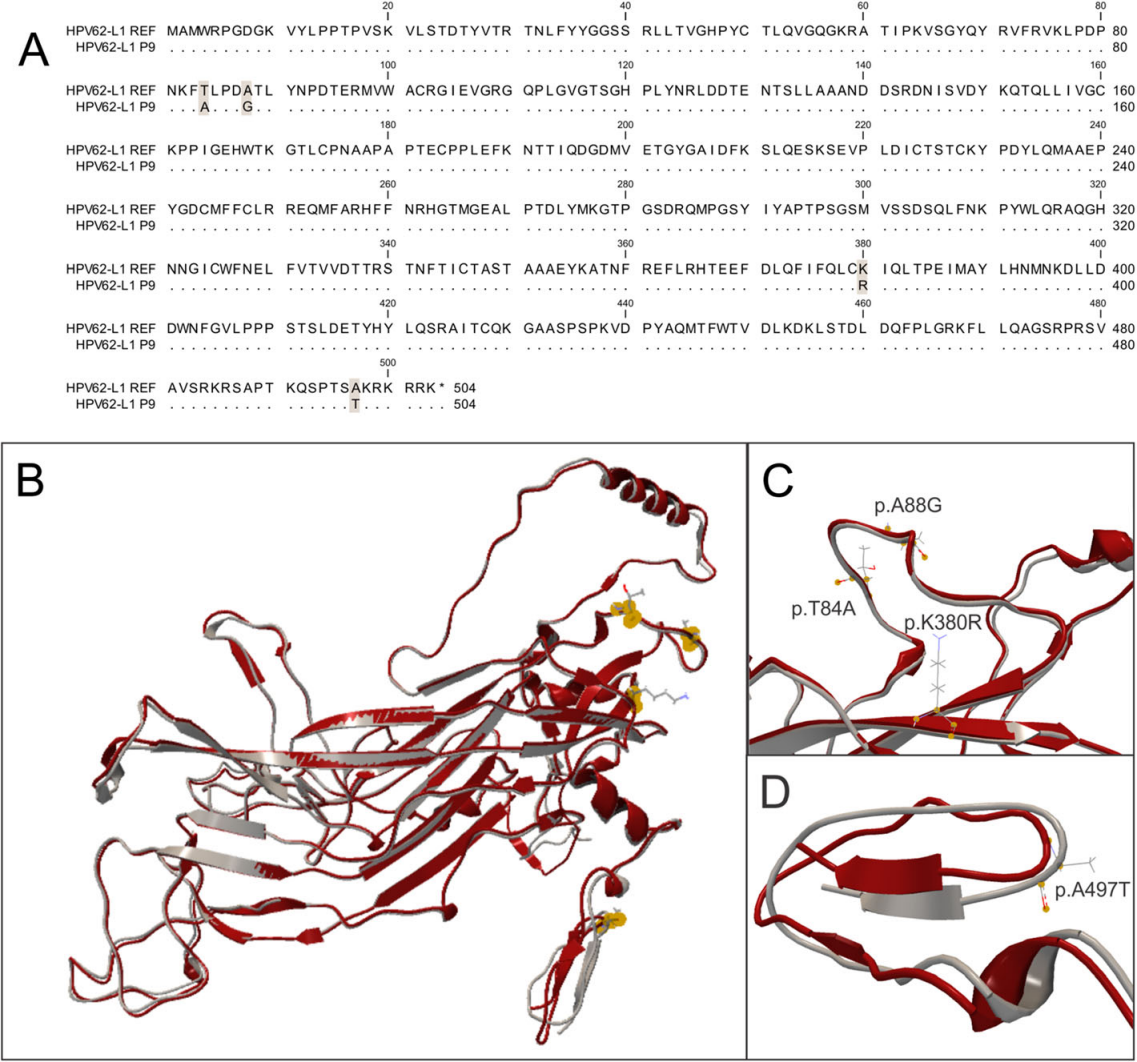
Sequence and structure alignment of HPV62 L1 protein. **a** Protein sequence alignment of the AY395706 NCBI sequence (HPV62-L1 REF) with that obtained from cervical sample P9 (HPV62-L1 P9); dots indicate matching residues, amino acid changes are darkened. **b** Structure alignment of both 503 amino acid complete proteins (HPV62-L1 REF in red and HPV62-L1 P9 in grey) and location of the four detected mutations shown as *yellow dots*. **c** Magnification of the structure alignment region containing the mutated amino acids threonine (p.T84A), alanine (p.A88G), lysine (p.K380R); and **d**) alanine (p. A497T)

To study whether the HPV62 that infects the Mexican population is a variant of the virus, the whole conserved HPV62-L1 ORF (1690 bp amplicon) from genomic DNA of a cervical sample was amplified and sequenced (HPV62-L1 P9). A total of 17 nucleotide changes were detected in HPV62-L1: four non-synonymous c.250A > G (pT84A), c.263C > G (p.A88G), c.1139A > G (p.K380R) and c.1489G > A (p.A497T); and 13 synonymous c.111G > A, c.117C > T, c.126 T > C, c.324C > T, c.627C > T, c.663G > A, c.1014 T > C, c.1017G > A, c.1161C > T, c.1416A > G, c.1425 T > A, c.1443 T > C, c.1497A > C (Fig. 2a). The first 3 amino acid substitutions are in the same spatial region in L1, while the last substitution is in the C-terminal domain, as shown by protein structure prediction (Fig. 2b, c, d). None of the four amino acid changes affect the tertiary structure of the L1 protein according to the predicted models.

### Nucleotide variations in HPV62-E6 and HPV62-E7

**Fig. 3:**
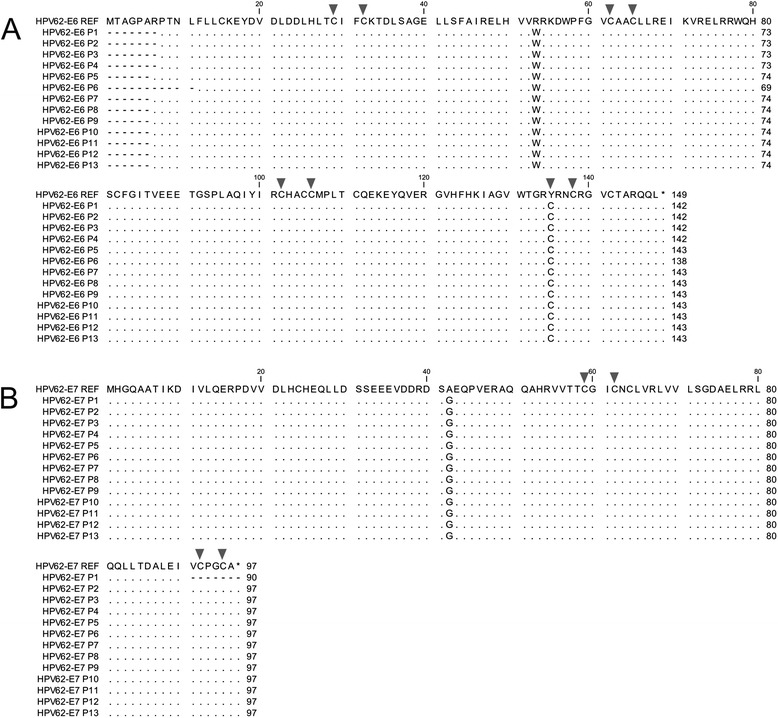
Protein alignment of HPV62-E6 and HPV62-E7. **a** Alignment of the HPV62-E6 reference protein sequence from AY395706 genome (HPV62-E6 REF) relative to the HPV62-E6 from 13 cervical samples (HPV62-E6 P1-P13). Amino acid changes p.R53W and p.K135C are shown. **b** Alignment of the HPV62-E7 reference protein sequence (HPV62-E7 REF) relative to the HPV62-E7 from the same 13 cervical samples (HPV62-E7 P1-P13). Amino acid change p.A42G is shown. Dots indicate matching residues and dashes indicate that no information is available for the corresponding region. *Arrows point* to cysteines that form the zinc binding domains

**Fig. 4:**
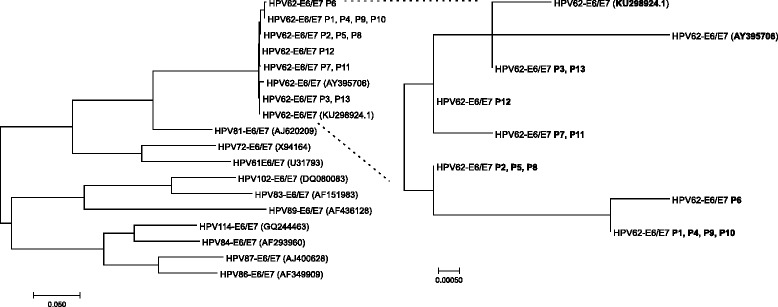
Phylogenetic tree showing reference HPVs from alpha-3 species and 13 HPV62 Mexican sequences based on E6/E7 genes. The evolutionary history was inferred by using the Maximum Likelihood method based on the Tamura-Nei model [37]. The tree is drawn to scale, with branch lengths measured in the number of substitutions per site. The analysis involved 18 nucleotide sequences. Evolutionary analyses were conducted in MEGA7 [38]. The GenBank accession number of each HPV genotype reference from alpha-3 species is included in parentheses

The E6 and E7 genes amplified from genomic DNA of 13 cervical samples infected with HPV62 were purified and sequenced to detect nucleotide variations in the HPV62 that circulates in Mexico. Altogether, seven nucleotide changes were identified in HPV62-E6, two of which were non-synonymous and led to an alteration in the protein sequence (c.157C > T, p.R53W and c.404A > G, p.Y135C). Regarding HPV62*-*E7, three nucleotide changes were detected and one of them affected the protein sequence (c.125C > G, p.A42G) (Table 4). The non-synonymous changes were present in all the samples, while the other nucleotide variations that do not change the amino acid were found only in some of them. The genetic sequences of E6 and E7 from HPV62 amplified from the 13 samples were translated into protein and aligned to the reference sequence for an easier visualization of the nucleotide changes and their location (Fig. 3). Finally, to visualize the relationship between all HPVs from Alpha-3 species and the HPV62 sequences obtained from the 13 cervical samples, a phylogenetic tree was built based on E6/E7 gene sequences (Fig. 4).

**Table 4:** Nucleotide changes found in *HPV62-E6* and *HPV62-E7* gene sequences from 13 cervical samples infected with HPV62 (P1-P13) compared to the reported HPV62 genome (AY395706, NCBI)

Mutations	P1	P2	P3	P4	P5	P6	P7	P8	P9	P10	P11	P12	P13
*HPV62-E6*													
c.27G > A	✓	✓		✓	✓	✓	✓	✓	✓	✓	✓	✓	
c.37 T > C	✓	✓		✓	✓	✓		✓	✓	✓			
c.157C > T*	✓	✓	✓	✓	✓	✓	✓	✓	✓	✓	✓	✓	✓
c.177 T > C							✓				✓		
c.199 T > C	✓			✓		✓			✓	✓			
c.201G > C	✓			✓		✓			✓	✓			
c.404A > G*	✓	✓	✓	✓	✓	✓	✓	✓	✓	✓	✓	✓	✓
HPV62-E7													
c.125C > G*	✓	✓	✓	✓	✓	✓	✓	✓	✓	✓	✓	✓	✓
c.183 T > C						✓							
c.199A > C	✓			✓		✓			✓	✓			

(*) Nucleotide substitutions that alter protein sequence

## Discussion

A fragment of the L1 ORF ─MY09/11 region─ of HPV62 was first sequenced in 1994 and the complete genome was isolated and sequenced to characterize the novel HPV type 62 in 2004 [13, 24]. In 2006, the Linear Array HPV Genotyping Test was launched; an assay that identifies up to 37 HPV genotypes in cervical samples, including HPV62. HPV62 belongs to the *Alphapapillomavirus genus*, species 3 group (α-3 group) together with HPV 61, 72, 81, 83, 84, 86, 87, 89, 102 and 114.

In the present study, from 578 HPV positive samples obtained from general population, CIN1 and CC women, the HPV62 frequency found with LA was 7.6, 8, and 4.6%, respectively, showing that this genotype has an important prevalence in Mexico; and it is mainly found in coinfection with HR-HPV genotypes (Table 1 and Fig. 1). Importantly, by using NGS, 11 out of 48 individually sequenced CIN1 samples were also positive for HPV62, despite only 8 of them were positive for this HPV genotype by Linear Array; moreover, concerning CC samples, only 1 of the 4 HPV62 positive samples detected by NGS, was positive with the last methodology. Therefore, the frequency of HPV62 could be underestimated. It is worth mentioning that, because cervical cancer cells were collected from cervical swabs and not from biopsies, the samples may contain normal cells together with cancer cells, so this could be a limitation of this study, since it is not possible to know which cells each HPV is infecting.

When HPV62 was characterized, it was one of the ten most prevalent HPV types detected in women with normal Pap smears from Costa Rica and New Mexico [13]. Later, more studies supported this finding. In the Italian population, HPV62 was detected in 1.5% of high-grade squamous intraepithelial lesions and also in 1.5% of CC, being the only LR-HPV found in CC samples [25]. There was no HPV62 detection in samples without cervical lesions in both populations. Among LR-HPV types detected in the Northern Indian population, HPV62 was the most common (10.5% of HPV positive samples) and in Egypt HPV62 was also the most prevalent LR-HPV among HPV positive women (17.4 and 9.7% in two different research works) [26–28]. In Thai women, HPV62 is also among the most frequent LR-HPV (11.3% of HPV positive samples) [29]. Another interesting result was found in females from the United States where the most common HPV type was HPV62 (found in 6.5% of all the subjects, where 42.5% are HPV positive) [30]. In Croatian women, HPV62 was among the most prevalent LR-HPVs in women with abnormal cervical cytology (23.3%) [31]. In Northeast Brazil, HPV62 prevalence among the overall population was of 3.6% and in Korea, 2% of atypical squamous cell and low-grade squamous intraepithelial lesions were HPV62 positive [32, 33]. In Mexico, a IMSS Research Network report on HPV, including 822 women, found HPV62 infection in 3.1% of women without cervical lesions, in 5.1% with CIN1, in 6.7% with CIN3 and in 0.8% in CC samples [14].

All these studies agree in the high frequency of HPV62 in cervical samples and its omnipresence in all kind of diagnosed samples; it is therefore of great interest to further study this genotype.

This work describes novel nucleotide changes in the HPV62-L1 complete gene (1512 bp). The 17 variations identified in the L1 ORF amplified from one sample determine a difference of 1.12% (17/1512 bp) compared to the reference sequence. A nucleotide sequence difference of 1% or more would define a new variant lineage, but according to the recommended new HPV variant classification criteria, the complete genome (and not only the L1 ORF) has to be sequenced [10]. The alignment of 20 HPV62-L1 sequences obtained by NGS using PGMY or FAP primer pairs from 16 cervical samples revealed the presence of a total of 19 mutations, 8 of them being non-synonymous. Interestingly, to our knowledge, only 3 out of the 19 mutations have been previously described [34]. It is worth mentioning that concerning those nucleotide changes, there was not a different distribution between CIN1 and CC samples, the mutations were distributed randomly among them.

Concerning HPV62-E6 and -E7 ORFs, the presence of genetic variations is described for the first time in the present study. Specifically, concerning HPV62-E6, non-synonymous nucleotide changes c.157C > T (p.R53W) and c.404A > G (p.Y135C) were found; additionally, c.125C > G (p.A42G) substitution in HPV62-E7 was found in all the 13 samples under study. However, the c.404A > G (p.Y135C) substitution that leads to a change of a tyrosine by a cysteine in all the 13 analyzed samples is located in a key position essential for the formation of a zinc binding domain which needs two CxxC motifs. Indeed, eight cysteines involved in the formation of two zinc binding sites in E6 protein are conserved among the different HPV types and the seventh cysteine in HPV62 is located in position 135, where a tyrosine was reported instead [12, 35]. HPV62 was originally characterized with the overlapping PCR method and according to the described methodological process, PCR products were visualized with UV illumination in agarose gels before product purification, ligation into pGEM-Teasy vector and sequencing. UV radiation is a strong mutagen that can induce conversion from one base to another [36]. Therefore, the adenine reported in position 404 of the reference genome could probably be due to a spontaneous change caused by UV radiation. The reference AY395706 HPV62 genomic sequence could have been reported with some mistakes and the three non-synonymous changes in E6 and E7 described in this work might be found of the HPV62 sequence worldwide. Earlier this year, a new complete HPV62 genome sequence has been uploaded in the GenBank database (KU298924.1) [15]; in which some of the nucleotide changes described in the present research are confirmed.

These findings open a new door to the study of LR-HPVs commonly found in HPV positive women. To date, oncogenic HPVs have been studied primarily, thus information on rare or LR-HPVs is limited, although they are often more prevalent than HR-HPVs. When found in coinfection with other HPV types, these non-oncogenic viruses may play an important role in the progression or regression of a cervical lesion, but this remains unstudied. Undoubtedly, more research is needed in this field, particularly with respect to the E5, E6 and E7 proteins and their different domains shared between HR-HPVs and LR-HPVs to understand their way of action. Furthermore, the L1 major capsid protein and its different variants may influence crucial steps of the viral infection cycle due to altered affinity to other proteins.

HPV infection in a single patient often comes along with more than one HPV genotype. Hence, clustering of the different genotypes present in a single sample might be crucial for understanding the roles of each genotype in carcinogenesis. This will lead to a better understanding of possible interactions between HPVs found in coinfections, both low and high risk.

## Conclusions

HPV62 was found in Mexican women who attended their preventive routine check-up and in women with cervical cancer. In the general population and in the CIN1 samples, HPV62 was often present both in single and multiple infection; however, in cervical cancer samples it was only found in coinfections with at least one HR-HPV type. To our knowledge, this is the first study that describes the presence of mutations in HPV62-E6 and -E7 genes. Moreover, newly observed nucleotide changes in the L1 gene were found to alter the L1 protein sequence. Upcoming discoveries in this field will complement the current information on variants of human papillomavirus and on still unclassified genotypes in their carcinogenicity risk to humans.

## Abbreviations

CC: Cervical Cancer
CIN1: Cervical Intraepithelial Neoplasia Grade 1
HR-HPV: High Risk Human Papillomavirus
LR-HPV: Low-Risk Human Papillomavirus
NGS: Next-Generation Sequencing
ORF: Open Reading Frame
